# Co‐Designing Recipe Resources to Support Healthy Eating in African‐Caribbeans in the United Kingdom: An Academic and Community Partnership Approach

**DOI:** 10.1111/jhn.13412

**Published:** 2024-12-19

**Authors:** Tanefa A. Apekey, Sally G. Moore, Maria J. Maynard

**Affiliations:** ^1^ Sheffield Centre for Health and Related Research (SCHARR) Sheffield UK; ^2^ School of Food Science and Nutrition University of Leeds Leeds UK; ^3^ School of Health Leeds Beckett University Leeds UK

**Keywords:** African‐Caribbean, co‐design, community, nutrients, recipe card, recipe modification

## Abstract

**Introduction:**

The wealth of free food‐based resources available to UK consumers on healthy eating and nutrition provides very limited illustrations of ethnic foods including African‐Caribbean cuisines. This inequality in available resources limits the ability of African‐Caribbean communities to effectively manage their health and reduces the cultural competence of health professionals.

**Objective:**

The aim was to co‐design healthier versions of several traditional African‐Caribbean recipe resources by working in partnership with academics, a community‐based Third Sector organisation, and their service‐users.

**Methods:**

Nutritional analysis software was used to theoretically modify the nutritional composition of popular traditional African‐Caribbean recipes using recently produced analytical food composition data. Twelve recipes were theoretically modified to reduce the content of key nutrients and ingredients of concern (i.e., salt/sodium, free sugars), or increase those nutrients known to be at risk of lower than adequate intakes (i.e., iron, folate) within the UK African‐Caribbean communities. Recipes were then prepared by community service‐users (*n* = 12) of African‐Caribbean ethnicity living in Leeds (UK) in the community service setting. The feasibility and acceptability of the recipes were evaluated by obtaining verbal feedback from service‐users, following which recipes were further refined as appropriate.

**Results:**

Modification resulted in a reduction in the overall energy (in the range of 23–188 kcal), fat (in the range of 0.1–13.7 g), saturated fatty acid (in the range of 0.1–2.9 g) and sugar (in the range of 0.2–8.3 g), provided by 100 g of the standard recipes. Similarly, modification resulted in the reduction in salt from about 63 to 0.01 g per 100 g edible portion of the standard recipe.

**Conclusion:**

It is feasible to modify African‐Caribbean recipes to be healthier and acceptable to consumers. Combined with improving access to food environments that make available healthy foods, the recipes are intended to support healthier eating with African‐Caribbean foods.

## Summary

- Our modified traditional recipes are evidence‐based and underpinned by reliable analytical food composition data.
- It is feasible to modify traditional African‐Caribbean recipes to be healthier and acceptable to consumers.
- Codesigned healthier traditional recipes could advance cultural competence in nutrition and dietetics practice, and would increase the healthy food options available to the UK population, further contributing to health promotion and reduction in health inequality.

## 1 Introduction

There are 18.3% of people other than White British ethnicity living in the United Kingdom, including 2.5% who identify as Black African (people of direct Black African origin) and 1.0% as African‐Caribbean (Caribbeans of Black African descent) [1]. Unfortunately, people of Black African and African‐Caribbean ethnicities in the United Kingdom continue to experience substantially poorer health outcomes and access to and use of health services compared to the White populations [2, 3, 4, 5, 6]. Excess weight, cardiovascular diseases, type 2 diabetes and associated behavioural risk factors such as physical inactivity, are more common in some minoritised ethnic groups compared to Whites in the United Kingdom [6, 7, 8]. It is well documented that various socio‐economic inequalities, structural racism and discrimination are major contributing factors [5, 6, 9].

Aimed at supporting people to improve health outcomes via diet through education, skill development and behavioural change, various nutrition and health promotional interventions have been developed and reported to date [10, 11]. For example, in the United Kingdom, there are several major ongoing public health initiatives to promote healthy eating, including salt, sodium, sugar and saturated fat reduction, and increasing fruit and vegetable (i.e., 5‐A‐Day) and fibre intakes [12]. These are associated with a wealth of free ‘tips’, recipes, ‘food swaps’ and other food‐based guidance resources, aimed at supporting people in the United Kingdom to eat healthier diets [13, 14]. Such resources, including ‘healthier’ recipes, are already known to be a key part of effective interventions that support the development of cooking skills and enable healthier eating in specific low‐income populations [15] and are used with people from diverse ethnic backgrounds [16]. In the United Kingdom, however, there are a very limited number of culturally adapted resources including recipes that reflect the characteristics, cultural customs, experiences, norms and beliefs of minoritised ethnic groups [17]. Such adaptation may, in the case of recipes, for example, incorporate specific traditional ethnic foods as part of healthier diets, including those commonly consumed in traditional African and African‐Caribbean cuisines.

Cultural adaptation in health promotion has been shown to increase the intervention's cultural ‘sensitivity’ and its effectiveness on the diet and health outcomes of participants [18]. Little insight into the development and performance of culturally adapted healthy eating interventions in the United Kingdom is available. Emerging evidence shows that cultural tailoring of nutrition education led to greater knowledge and long‐term diabetes management improvements, compared to usual care [19, 20]. Furthermore, the need for such culturally adapted resources to diversify healthy eating and nutrition advice has recently been professionally recognised by dietitians in the United Kingdom [21]. The continued absence of culturally tailored healthy eating advice and resources could negatively impact care communication (patient and provider) with health professionals, assessment and nutrition‐related outcomes for African‐Caribbeans in the United Kingdom [22].

Another way to ensure that health interventions are developed to be more acceptable and effective for the target population is via a ‘co‐design’ process, defined as ‘where people with professional and lived experience partner as equals to improve health services by listening, learning and making decisions together’ [23]. Such approaches that engage the community are increasingly being used to culturally adapt healthy‐eating‐related interventions [24], including those in the United Kingdom developed to support healthier eating and management of type 2 diabetes amongst people of self‐defined Black‐British, Black African or Black Caribbean ethnicities living in London, UK [20]. Partnership working across communities, intended end‐users, staff and researchers can facilitate meaningful connections and dialogue to further understand service‐users' health and cultural needs [24]. In addition, partnership working has the potential to ensure health equity, especially in Black minoritised ethnic groups who are under‐represented in health research and professional practice [25, 26, 27]. Overall, there is a current need for further research involving co‐design and cultural adaption, leading to the evaluation of the effectiveness of such interventions on health and nutrition in the target populations [28].

The aim of this work is to co‐design and develop culturally appropriate healthier recipe resources, which are aimed at, and acceptable to, people of UK African‐Caribbean ethnicity. The work was made possible by a new partnership between nutrition academics, a Third Sector community organisation and service‐users of African‐Caribbean ethnicity living in Leeds, UK. The specific objectives were to nutritionally modify standard African‐Caribbean recipes that are made using traditional ingredients and to establish their feasibility and acceptability among staff and service‐users at the Third Sector community organisation, particularly people of African‐Caribbean ethnicity.

## 2 Methods

### 2.1 Aims and Approach

A recipe resource development study was conducted using a co‐design approach for which a new working partnership was set up between the researchers (T.A.A., S.G.M. and M.J.M.), a staff and service‐users at a community‐based health social care Third Sector organisation in Leeds, UK. The Third Sector partner supports residents of Chapeltown and Harehills local areas in Leeds. Chapeltown and Harehills are among the fourteen lower super output areas (SOA) of Leeds and have very diverse populations, including people of African‐Caribbean ethnicity [29]. SOA are hierarchical classification of geographic areas in the United Kingdom based on population size and various socio‐economic and demographic characteristics. UK Third Sector health and social care community organisations (also referred to as the community and voluntary sector) provide a range of health services and opportunities, especially in socio‐economically deprived communities that lack health equity. Thus, a community‐based approach and Third Sector partnership are important and have the potential to make a difference to the local community. This approach and partnership will also facilitate effective cultural competence and health promotion in the target community.

Eligible participants were adults at least 18 years old of self‐defined African‐Caribbean ethnicity living in Leeds. Potential participants were recruited by convenient sampling from existing service‐users by staff at the Third Sector partner. Staff informed service‐users about the study at their group sessions following which 12 expressed an interest. Each potential participant received an information sheet prior to the commencement of the study and those interested signed a consent form, in accordance with the 1975 Declaration of Helsinki. Upon completion of the study, service‐users received a £25 shopping voucher as an appreciation of their time.

**Figure 1:**
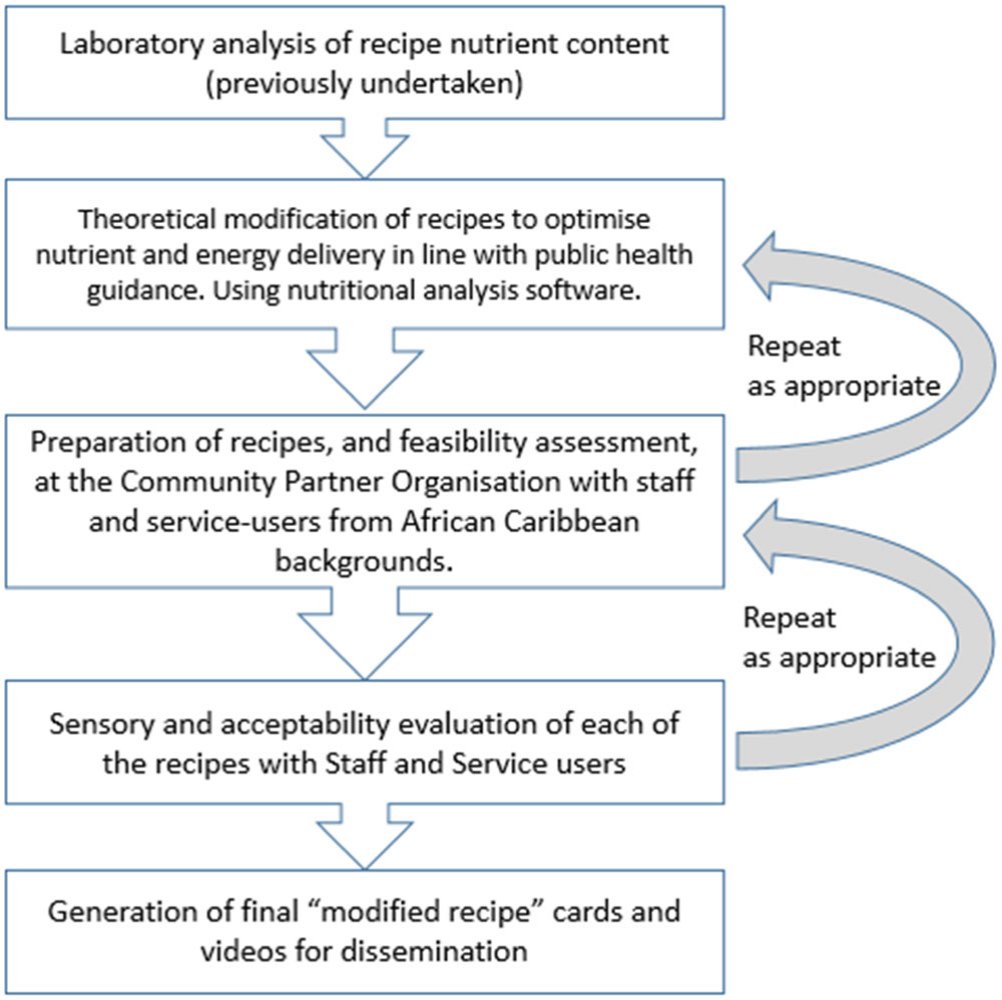
Flow chart to illustrate the recipe modification and co‐design process undertaken to produce feasible and acceptable healthier versions of traditional African‐Caribbean recipes.

Recipe modification stages included the use of theoretical nutrition analysis by the researchers and student volunteers, followed by cooking/preparation and tasting by the service‐users, held at the community partner organisation (Figure 1).

All 12 had cooking skills and participated in the 5 cooking sessions and consumed what they prepared before providing feedback. The cooking sessions were facilitated by a Third Sector staff of African‐Caribbean ethnicity, supported by the researchers and students. In this co‐design approach, multiple stages of recipe theoretical nutritional composition calculations, modifications to ingredients and cooking methods, and theoretical evaluation of nutrient and energy delivery were used. Cooking/testing and refinements were then conducted with people of African‐Caribbean ethnicity to ensure that the modified recipes (MR) were culturally acceptable and relevant to them.

### 2.2 Recipe Resource Development

#### 2.2.1 Selection of Traditional Recipes and Their ‘Healthier’ Modifications

Several traditional African‐Caribbean recipes and foods/ingredients have previously been selected for nutritional analysis, based on their popularity and consumption in the United Kingdom [30]. Nutrient and energy delivery of these had thereafter been established via ingredient‐accredited laboratory analysis of the selected foods. The description and analytical nutrient composition of the African‐Caribbean traditional foods modified are outlined in Apekey et al. [31]. This information was used as the starting point on which to calculate nutritional composition data for whole recipes and evaluate the potential for modifications to these in order to provide ‘healthier versions/recipe modifications’ (i.e., via the use of ingredient alternatives and cooking method changes, Table 1). This was done with the following aims corresponding to UK public health nutrition advice and food‐ and nutrient‐based guidance reflecting Government Dietary Recommendations [32] and the nutritional needs of the target population [6].

**Table 1:** Summary of modifications to the ingredients and cooking methods of each recipe that support healthier eating.

Type of food	Name of recipe	Summary of main modifications to the standard recipe
Beverage	Rum punch	Alcoholic rum and syrup replaced with rum essence and a variety of fresh fruits.
Snack	Meat patties	White flour, butter and minced meat replaced with whole meal flour, lean mincemeat (5% fat) and low‐fat spread (26%–39% fat).
Breakfast	Cornmeal porridge	Sweetened condensed milk and white sugar taken out of the recipe.
Dish	Jerk chicken	Chicken replaced with skinless chicken and the use of freshly made jerk spice.
Dish	Ackee and saltfish	Saltfish soaked overnight to reduce salt content and freshly made jerk spice.
Dish	Callaloo and saltfish	Saltfish soaked overnight to reduce salt content.
Dish	Fish curry	Coconut milk and butter replaced with reduced fat coconut milk (30% less fat) and olive oil spray.
Dish	Goat curry	Coconut cream replaced with reduced fat coconut milk (30% less fat).
Dish	Vegetable curry	Coconut milk and butter replaced with reduced fat coconut milk (30% less fat) and olive oil spray (0.1 g fat per spray).
Dish	Rice and peas	Coconut milk replaced with reduced fat coconut milk (30% less fat).
Dish	Saltfish fritters	White flour replaced with wholemeal flour. The fritters were baked in place of deep fat frying.
Dish	West Indian soup^a^	Meat^b^ and white flour (for the dumplings) replaced with skinless chicken and wholemeal flour, respectively.

^a^ Soup of thick consistency.

^b^ Offcuts of beef (e.g., beef shin) and other meat (e.g., mutton, lamb).

Recipes were theoretically modified, using *Nutritics* (utilises UK food composition database) nutritional analysis software (https://en-gb.nutritics.com/p/home) to evaluate the content of energy, saturates, sugars, salt/sodium, fibre, fruit and vegetables with respect to the UK Dietary Recommendations for these nutrients/components (see bullet points below). In addition, traffic light colour coding was also used to evaluate nutrient delivery per 100 g and per serving (adult) of the edible portion of each recipe and target nutrients for reduction (i.e., salt/sodium, saturated fat, sugars) [33], with the intention of providing this information on the final recipes.

- Achieve energy balance, with a reduction in calorie intake where appropriate, to manage healthy body weight.
- Reduce salt and sodium (< 6 g per day; equivalent to < 2.4 g of sodium).
- Reduce saturated fat (< 10% of energy intake, replace with monounsaturates).
- Reduced sugar (< 5% of energy intake from free sugars).
- Increase fruit and vegetables (achieve at least five portions of fruit and vegetables per day).
- Increase fibre (30 g/day, adults).

Preserving the authenticity of the MR involved no introduction of new ingredients but rather enhancing traditional ones like fruits and vegetables, and fresh herbs, while also decreasing those that contribute to salt, sodium, saturated fatty acids, total fat and free sugars found in the standard recipes (SR). Further details are provided in Table 1.

#### 2.2.2 Recipe Testing and Refinement

A total of twelve women, first and second generation African‐Caribbean staff and service‐users from the Third Sector healthy living centre in Leeds, partnered with the research team to co‐design the healthier recipe resources. The cooking sessions (*n* = 5) were interactive and lasted for about 3 h each. Ingredients for the recipe preparations were purchased locally from ethnic food retailers in the community. The MR were then prepared by the staff (*n* = 1, facilitator of the cooking session) and service‐users (*n* = 12) in the healthy living centre's kitchen. During the food preparation stage, further refinements were made (where appropriate) to recipes and food preparation methods based on suggestions from the service‐users (e.g., increasing the quantity of chilli pepper) to ensure ‘feasibility’.

The prepared recipes were then consumed, and a short evaluation form was used to obtain qualitative feedback (subjective evaluation) on key sensory attributes (i.e., appearance, taste, texture and flavour) and ‘acceptability’, from the staff and service‐users. The purpose of the qualitative evaluation was to obtain feedback on suggestions for recipe refinement, acceptability and feasibility and not patterns of meanings. Hence, thematic analysis was not required. Also collected by the researchers were brief notes on participants' verbal feedback. These evaluations were synthesised by researchers and led to further refinement of some recipes in line with healthy eating recommendations (e.g., suggestion of the alternative option of 50/50 self‐raising flour for wholemeal flour in the case of the modified dumplings). Photographs of the prepared dishes were taken for the preparation of the recipe cards. Cookery videos of the recipe preparations were also recorded and are currently being edited.

#### 2.2.3 Nutritional Analysis of Refined Recipes and Development of Recipe Cards

The refined recipes were then analysed by Apekey and Moore (who supervised student research volunteers) using *Nutritics* software (https://en-gb.nutritics.com/p/home). Any differences in nutrient composition were discussed (e.g., agreement on the choice of similar ingredients) and resolved by consensus. The information presented on each MR card are:

- Real images of the prepared dishes, snacks and beverage
- Adult serving portion suggestion
- Ingredients and their quantities
- Food preparation method and duration, and
- Nutrition information per 100 g of edible portion and traffic light labelling (information per adult serving portion).

Due to the absence of reliable data on typical serving portions of traditional African‐Caribbean dishes, those of similar dishes, snacks and beverages available in UK supermarkets were used in the preparation of the recipe cards.

### 2.3 Community‐Based Public Engagement Event

As an appreciation of the partnership, to promote and share the MR and encourage participation in health opportunities and research, the team delivered a free community‐based health and nutrition awareness‐raising event in Leeds. The offerings at the event included health checks (weight, height, body mass index, waist circumference, blood pressure, cholesterol etc) and advice, MR cards and a selection of the prepared MR, and verbal and written feedback was also obtained from attendees.

This study was conducted according to the guidelines laid down in the Declaration of Helsinki and all procedures involving research study participants were approved by the Leeds Beckett University Research Ethics Committee, Ref: 99311. Written informed consent was obtained from all subjects.

## 3 Results

### 3.1 Recipe Cards

The new recipe cards of the modified African‐Caribbean recipes are accessible via this *link*: https://github.com/Mansavi3/Co-produced-Modified-Caribbean-Recipes-2024.git. Compared to the SR, the modified versions contained less calories and fat. Modification of the recipes used traffic light at theoretical nutritional analysis stages to identify ‘red’ (high) levels of specific nutrients and enable healthier quantities [change from red/to amber (medium)/green (low)]. In the current study, nine of the 12 recipes had green levels of calories, fat, SFA, sugars and salt. Sodium is not declared in the UK traffic light labelling system because salt is deemed more readily comprehensible by consumers. With regards to the modified callaloo and saltfish, and meat patties recipes, fat content changed from red to amber traffic light without becoming unacceptable to participants.

### 3.2 Nutritional Composition and Recipe Cards

#### 3.2.1 Comparison of the Nutritional Information (Per 100 g Edible Portion; Per Average Serving Size) of Standard and MR

**Figure 2:**
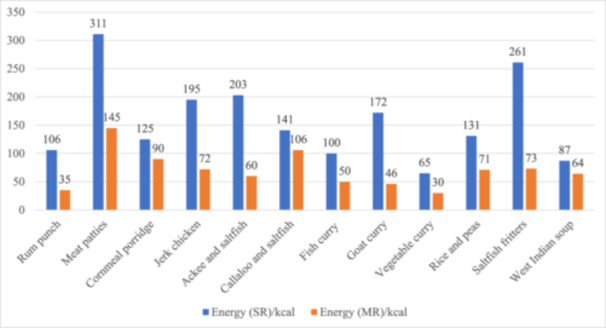
Comparison of the energy (kcal) content per 100 g of edible portion of the standard (SR) and modified (MR) recipes.

**Figure 3:**
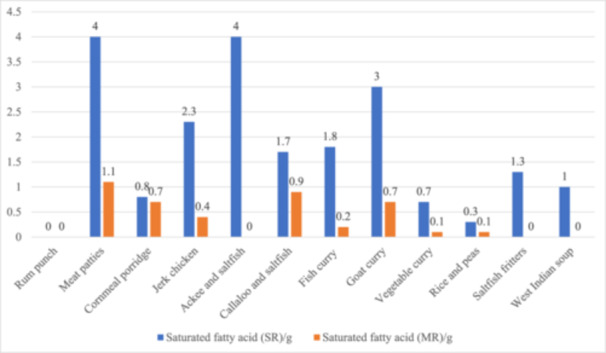
Comparison of the SFA (g) content per 100 g of edible portion of the standard (SR) and modified (MR) recipes.

**Figure 4:**
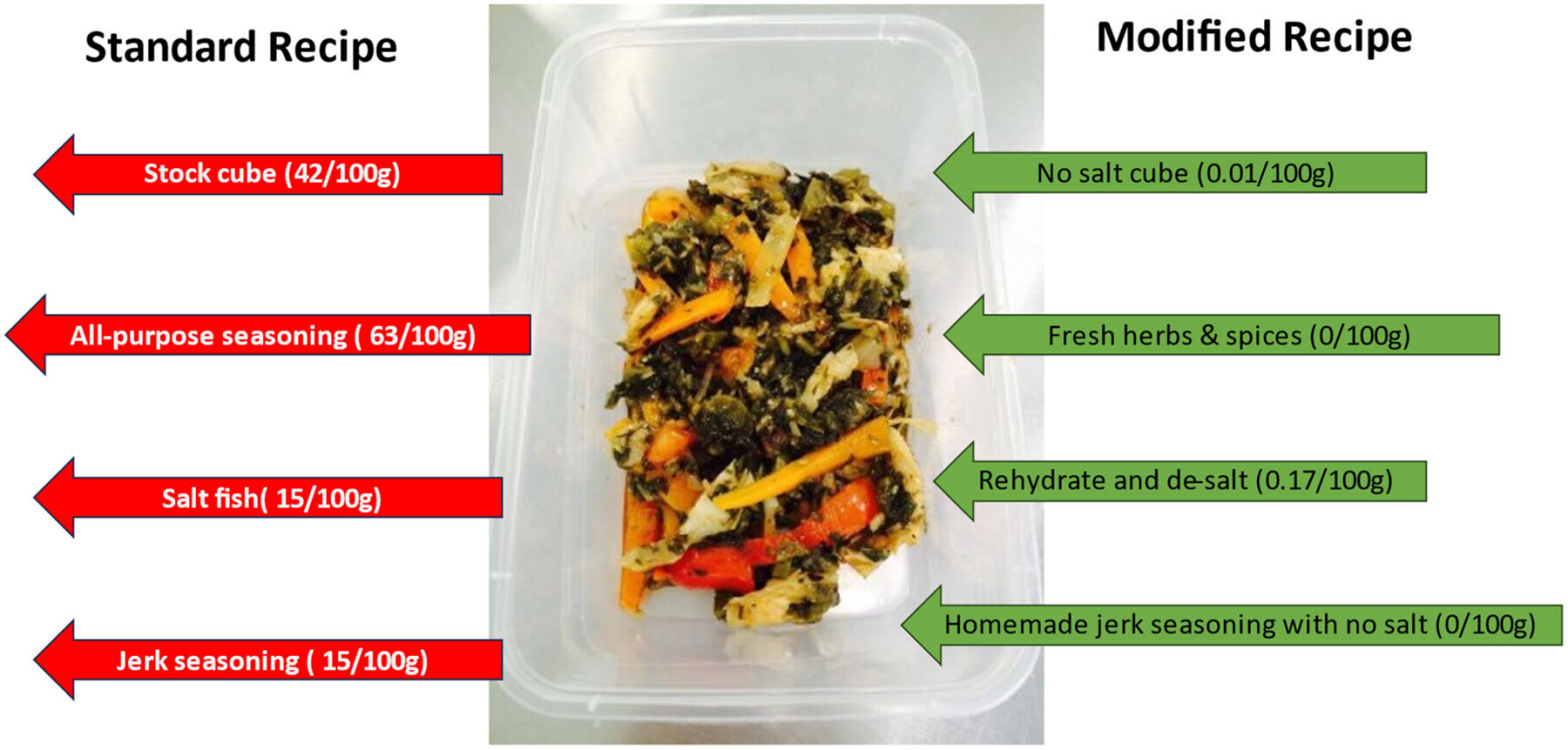
Illustration of the approach to salt reduction in the standard recipes.

A comparison of the energy and SFA composition per 100 g edible portion of the SR and MR is shown in Figures 2 and 3, respectively. Figure 4 illustrates the reduction in the salt content of the recipes.

Changes in the carbohydrates, sugars, dietary fibre, protein and fat levels of the recipes are shown in Supporting Information S1: Figure S1,Supporting Information S2: Figure S2, Supporting Information S3: Figure S3, Supporting Information S4: Figure S4 and Supporting Information S5: Figure S5. Modification (mainly a reduction in total fat Supporting Information S5: Figure S5 and sugar Supporting Information S2: Figure S2) resulted in a 23–188 kcal reduction in the overall energy provided by all the SR. Apart from cornmeal porridge, modification led to a 0.1–13.7 g reduction in the total fat, and 0.1–2.9 g decrease in the SFA content across all the recipes. In addition, the majority of the MR contained less sugar (0.2–8.3 g, Supporting Information S2: Figure S2) compared to the standard versions. The salt‐containing ingredients were stock cube, All‐purpose seasoning (dry), Jerk seasoning (wet), and salted fish (dry). Figure 4 illustrates the reduction in salt content achieved via alternative ingredients. Ingredients shown in red were taken out of the SR and replaced with those in green. The modified rum punch and cornmeal porridge recipes (Supporting Information S2: Figure S2) showed the most significant reduction in sugar (8.3 and 7.9 g, respectively). This was achieved by substituting alcoholic rum with rum essence and sweetened condensed milk with fresh semi‐skimmed milk, respectively. The change in protein (Supporting Information S4: Figure S4) and SFA (Figure 3) content is mostly due to the use of lean protein such as skinless chicken for the modified jerk chicken.

**Figure 5:**
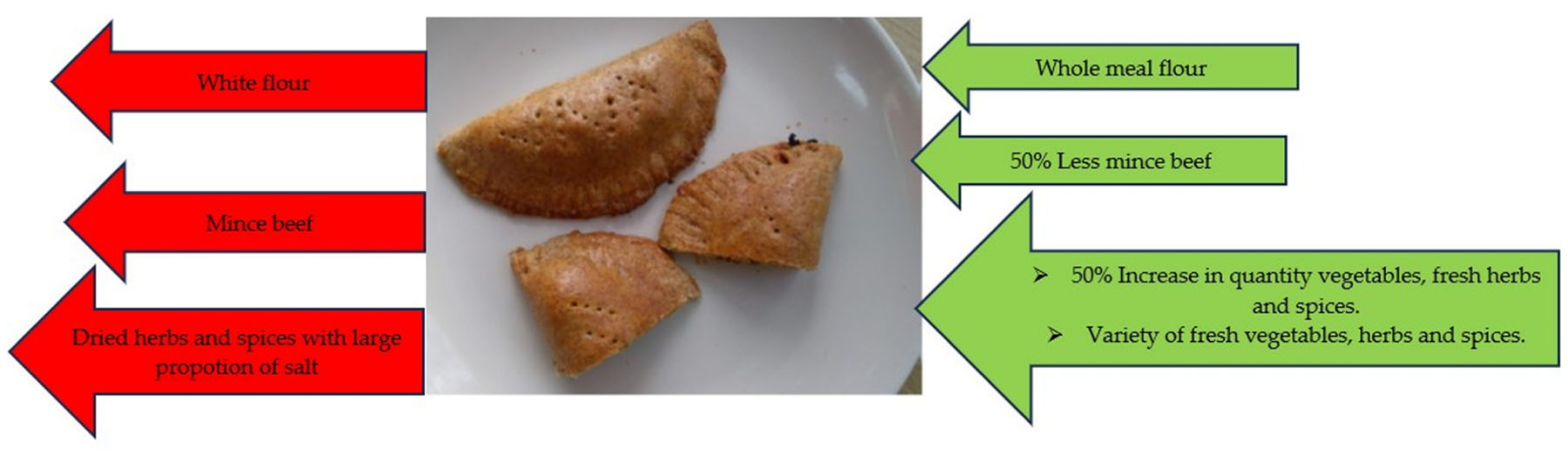
Illustration of the approach to fibre increment in the standard recipes.

A comparison of the average portion size of SR and MR (Table 2) showed similar changes in energy, fat, SFA, carbohydrate, sugars, protein and fibre. An increase in fibre was achieved through the use of a variety and increased proportions of fresh (or frozen) fruits, vegetables, herbs and spices, as illustrated in Figure 5. Thus, in most cases, modification resulted in an increase in fibre content, and fruit and vegetable portions (were relevant) relative to standard versions.

**Table 2:** Comparison of the nutritional information per average serving size of standard and modified recipes.

Food name (average serving size)	Energy SR (kcal)	Energy MR (kcal)	Fat SR (g)	Fat MR (g)	SFA SR (g)	SFA MR (g)	Carbohydrate SR (g)	Carbohydrate MR (g)	Sugars SR (g)	Sugars MR (g)	Fibre SR (g)	Fibre MR (g)	Protein SR (g)	Protein MR (g)
Ackee and saltfish (200 g)	406	120	32.2	4.8	8	0	7	9.2	7	7.4	4.6	3.2	19.6	8
Callaloo and saltfish (200 g)	282	211	18.8	6.3	3.4	1.9	8.6	24	8.6	10	6	3	16.6	13.2
Cornmeal porridge (197 g)	246	178	2.6	2.6	1.6	1.4	47	30	21.3	5.8	1.8	1.6	7.7	7.1
Fish curry (200 g)	200	100	8.6	1	3.6	0.4	7.2	7.2	7.2	4.2	6	2	20.6	7.2
Goat curry (200 g)	344	91	20.4	3	6.2	1.4	< 0.1	5.8	3.8	3.2	4	1.4	37.4	7.8
Vegetable curry (200 g)	130	60	5.6	1	1.4	0.2	14.8	8.4	10	6	2.2	3.8	4	2.4
Jerk Chicken (250 g)	488	181	24	3.7	5.8	0.9	< 0.1	5	5.5	1.7	0	1.25	68.5	32.5
Meat patties (150 g)	467	217	18	5.8	6	1.6	56.1	24	5	3.9	4.5	1.8	17.9	16.5
Saltfish fritters (125 g)	326	92	16.9	0.6	1.6	0	30.1	13.8	3	3.1	2.8	1.25	11.8	7
Rice and peas (125 g)	164	89	1	0.5	0.4	0.1	31	17.8	0.9	1.3	3.6	2	6.1	3.3
Rum punch (250 g)	265	89	0.3	0.5	< 0.1	0	65.3	16.8	12.3	16	0.2	1.3	0.3	1.5
West Indian soup (300 g)	261	64	7.2	1.4	3	0.2	29.7	28.5	6.6	6.4	6	1.2	16.2	14.7

### 3.3 Qualitative Feedback on the MR

All the new MR were positively evaluated by the partners. Each recipe was tested once since the suggestions for refinements were few, minor and based on spice preference, for example, add more scotch bonnet pepper. Such suggestions were included in the recipe card so people could adapt to suit their taste preferences. The ‘acceptability’ and ‘feasibility’ of the MR are reflected in the selected quotations in Table 3. Suggestions for further refinement of the recipes are also indicated.

**Table 3:** Service‐users' qualitative evaluation of the modified recipes.

Beverage/dish/snack	Quotation
Modified rice and peas	‘There is nothing there for us, if we can get this in hospital it will be alright’ ‘Very nice’
Modified West Indian soup	‘Good colour’ ‘Tastes good’ ‘Pumpkin too soft’ ‘Use chicken thighs’ ‘Wash chicken thighs with vinegar or lemon juice’ ‘Never had it before but ate it all’ ‘Still tasty’ ‘Ate it all’ ‘Wholemeal dumplings just as nice’
Modified cornmeal porridge	‘Add more vanilla’ ‘It is sweeter’ ‘Not had it before’ ‘Nice’ ‘Would put honey at home’ ‘Doesn't need sugar, can be sweet or savory’ ‘Texture is good’
Modified jerk chicken	‘Beautiful, tastes lovely’ ‘very nice but not too hot’ ‘Just nice, not too hot’
Modified rum punch	‘Good’ ‘Can taste ginger’ ‘like it’ good’ ‘less lime’ ‘doesn't normally add ginger but very nice’ ‘Really nice’
Modified vegetable curry	‘Lovely’ ‘Gorgeous’ ‘Beautiful’ ‘good’ ‘New vegan choice’
Modified ackee and saltfish	‘Add more sweet pepper of different colours’ ‘prefer softer texture’ ‘good’
Modified callaloo and saltfish	‘Very nice’ ‘Tastes the same’ ‘Good’ ‘Best thing is that it not too salty’ ‘Add more black pepper’ ‘Good’
Modified meat patties	‘A lot nicer than I thought’ ‘A little less salt’ ‘More pastry’ ‘Filling a bit dry’ ‘good’

Feedback from the Third Sector partner and community‐based health event attendees is also shown below.

Our service‐users really benefited from participating in this project. Not only did it help reflection on their own healthy eating and cooking habits, they enjoyed the discussion and learning as a group together. Our‐service users thoroughly enjoyed the opportunity to pass on their skills and share their traditional recipes.

The community‐based health event for sharing the resources and provision of health checks was attended by over 100 people, mostly of African‐Caribbean ethnicity. Overall, recipe cards and prepared recipes were well received [‘Food was really nice’ ‘Food was very nice’ ‘Food was enjoyable’ ‘Good food’ ‘Going to try some recipe’].

## 4 Discussion

Reliable and comprehensive food composition data is crucial for recipe reformulation and the creation of evidence‐based healthy eating resources to address health inequalities. Despite the growing popularity of ethnic foods, the UK nutrient database contains only a limited selection. The current reformulation project has been made possible thanks to the analytical food composition data for African and African Caribbean foods [30, 31] previously generated by the research team.

In the United Kingdom, generic health promotion resources, such as the Eatwell Guide and mass media campaigns, have been shown to be potentially ineffective at reaching diverse groups and socio‐economically deprived populations because they are not tailored to their needs and lifestyles [34, 35, 36]. This project is part of a wider research programme to produce a range of recipe resources to advance nutrition literacy and health promotion in people of African and African‐Caribbean ethnicities, and to build capacity for community‐based Third Sector organisations. Another aim of the overall research programme is to help support the cultural competence of Third Sector health professionals, Dietitians and Nutritionists who may use these co‐designed recipe resources when working in diverse and underserved communities. In the current study, the co‐designed recipe modifications resulted in substantial reductions in the total energy (up to 72%), fat (up to 85%), SFA (up to 72.5%), sugars (up to 55.7%) and salt and sodium (up to 95%) provided by the SR. In addition, the salt content of the MR was less than 1 g per 100 g edible portion, and per adult serving portion and therefore did not exceed the daily limits of less than 6 g. Furthermore, the MR evaluated very well in people from the target population. The results of the current study align with those of Stubenitsky et al. [37] and Patel et al. [38], who reported that recipe modification (reduction in fat, energy, SFA and sodium) did not affect restaurant meal acceptance. Thus, the MR have the potential to improve diet and nutrition, and to promote healthy weight in African‐Caribbeans.

High salt intake is associated with an increased risk of high blood pressure, which is increasingly more common in people of African and African‐Caribbean ethnicities compared to White groups in the United Kingdom [6]. Dietary advice for this population should promote replacing ‘All‐purpose seasoning’ and stock cubes with fresh traditional herbs and spices or salt‐free seasoning in cooking. The soaking of saltfish overnight to reduce the salt and sodium content or replacing it with unsalted cod or haddock fish should also be promoted.

Front‐of‐pack ‘traffic light’ nutrition labels are a current UK public health policy to promote healthy eating and are now widely available on food products sold in supermarkets. Alongside mandatory nutrition information, the front‐of‐pack traffic light is expected to enable consumers to see the content of specific nutrients (e.g., saturates) and ingredients (i.e., salt, sugars) of public health concerns in the food or beverage, to support healthier choices. The modification of standard African‐Caribbean recipes resulted in healthier levels of nutrients and ingredients. Thus, the provision of traffic light and nutrition information on the recipe cards reflects existing UK Government policy aimed at enabling consumers healthier choices and creating healthier food environments [33] and could (i) provide better understanding and motivation for healthier choices in the target population, (ii) facilitate cultural competence and better patient‐professional or service‐user/professional interaction and care communications, (iii) contribute to food‐based resources for public health interventions and health promotion activities in this population, and (iv) encourage individuals of other ethnicities to try new recipes, and therefore extend the healthier food options available to the UK public.

Demonstration cookery videos (e.g., YouTube videos) are becoming increasingly popular for many reasons, including the availability of portable devices that support videos, their use to enable people to develop cooking skills and interest in trying new recipes [39], and therefore have the potential to influence users' nutrition and diet quality. The Covid‐19 pandemic has also reshaped food and drink trends, with a rise in home cooking, a preference for healthier foods and the use of demonstration videos [40, 41].

Similarly, African‐Caribbean cuisines and cookery videos are increasingly popular in the United Kingdom [42, 43, 44, 45, 46]. Hence, the need to film the recipe preparations as an additional resource for promoting healthy eating in the target population and general public. The recipe cards are currently being shared and promoted through local authorities and Third Sector organisations (e.g., Modified Caribbean Vegetable Curry | FoodWise [foodwiseleeds.org]; Caribbean Rum Punch Mocktails‐ Heart Research UK). The cards are also being used in research interventions with researchers at the University of Sheffield (T.A.A.), Leeds Beckett University (M.J.M.) and University of Leeds (S.G.M.) to support healthy eating in the target population and cultural competence in health professionals. The project is ongoing, a range of cookery videos recipe cards and healthy eating applications for mobile devices are under development for sharing with relevant stakeholders and the public.

### 4.1 Strengths and Limitations

To the best of the researchers' knowledge, there is no evidence of co‐designed healthier African‐Caribbean recipe resources in the United Kingdom. The new recipe cards and further resources to be produced from this project could potentially contribute to a reduction in health inequality by advancing nutrition knowledge, encouraging home cooking where cooking is not often performed and adding to the food‐based recipe resources available to the UK public. The resources also have the potential to diversify nutrition and dietetics practice, highlighting the importance of this diversity in meeting the health needs of minoritised ethnic groups. This new collaboration was an enjoyable learning experience for all the partners. It, therefore, adds to the evidence that people of Black ethnicity are willing to participate in research, and co‐production (development) research with Third Sector organisations and African‐Caribbean service‐users can work well. This work also provides a framework on which to base further healthier recipe development, including via a co‐design process with members of the target population.

Although the MR were evaluated by volunteers from the relevant ethnic group, health professionals and the public, a scientifically robust evaluation is required to assess the reach, utilisation and effectiveness of the resources in promoting healthy eating at individual and household levels, as well as effectiveness at enhancing competent nutrition and dietetics practice. We also recognise that the volunteers do not represent the diversity of the UK Black African and African Caribbean population. Understanding the wider dietary and nutritional impact of these recipe modifications is crucial. We are currently collecting qualitative case studies from nutrition professionals and members of the relevant ethnic groups to inform a future impact assessment with a larger, representative sample. As with any nutrition analysis software, the accuracy relies heavily on the completeness and accuracy of the Nutrient database the software uses. The nutrition analysis software used in this project is based on the UK food composition database, which has very limited data on ethnic foods, including African‐Caribbean foods. Hence, approximates were used (i.e., similar food ingredients), in some instances, which could lead to inaccuracies and not reflect the real improvements made to the SR. This reflects the need [31] for the food composition databases to better reflect traditional foods and ingredients.

Maximising the reach, integration into services and utilisation of the recipe resources beyond Leeds depends on collaborative efforts and capacity building among stakeholders. To achieve this, we propose (and are currently implementing) the following strategies to engage stakeholders through local channels (i) disseminating our research findings and emerging stakeholder feedback through meetings, workshops and written materials, (ii) offering practical educational resources, such as short videos, community education, training workshops and cooking demonstrations, along with support for their delivery, (iii) Influencing policy processes, standards and guideline development by holding meetings with local authorities and policymakers to share findings and facilitate their integration into relevant policies to improve practice, and (iv) preparing policy briefs, advocating for cultural adaptations in public health messaging and e‐health initiatives (e.g., Digital Dietary Interventions) and (v) ongoing monitoring and stakeholder feedback to assess the effectiveness of these initiatives and inform future improvements.

## 5 Conclusion

It is feasible to use a co‐development partnership approach to modify traditional recipes to be healthier and also acceptable to those who are familiar with traditional African‐Caribbean foods. To ensure their acceptability, modifications retained the authenticity of SR by increasing the amounts of traditional vegetables, fruits, wholegrains, herbs and spices, while reducing the contributors of salt/sodium, saturated fatty acids, free sugars and dietary fat. The findings warrant future research extension to include a formal evaluation of the health‐related impact of the resources, in combination with new multicultural food‐based guidance, on the target communities.

## Author Contributions

T.A.A. designed the research. T.A.A., S.G.M. and M.J.M. conducted the research. T.A.A. and S.G.M. analysed the data. T.A.A., S.G.M. and M.J.M. wrote the paper. T.A.A. had the primary responsibility for the final content. All authors read and approved the final manuscript.

## Ethics Statement

This study was approved by the Leeds Beckett University Research Ethics Committee, Ref: 99311.

## Conflicts of Interest

The authors declare no conflicts of interest.

### Transparent Peer Review

The peer review history for this article is available at https://www.webofscience.com/api/gateway/wos/peer-review/10.1111/jhn.13412.

## Supporting information

Supporting information.

Supporting information.

Supporting information.

Supporting information.

Supporting information.

## Data Availability

The data that support the findings of this study are available from the corresponding author upon reasonable request.
