# Supplementary material for: Co‐Designing Recipe Resources to Support Healthy Eating in African‐Caribbeans in the United Kingdom: An Academic and Community Partnership Approach
Source: J Hum Nutr Diet. 2024 Dec 19;38(1):e13412. doi: 10.1111/jhn.13412 (PMC11659633; doi:10.1111/jhn.13412)
Supplement: Supplementary file 4 — Supporting information. [file JHN-38-0-s002.docx]

**Figure S4** Comparison of the **protein (g)** content per 100g of edible portion of the standard (SR) and modified (MR) recipes
